# Dissociable theta networks underlie the switch and mixing costs during task switching

**DOI:** 10.1002/hbm.25573

**Published:** 2021-06-29

**Authors:** Montana McKewen, Patrick S. Cooper, Patrick Skippen, Aaron S. W. Wong, Patricia T. Michie, Frini Karayanidis

**Affiliations:** ^1^ Functional Neuroimaging Laboratory, School of Psychology University of Newcastle Callaghan New South Wales Australia; ^2^ Priority Research Centre for Brain and Mental Health University of Newcastle Callaghan New South Wales Australia; ^3^ Priority Research Centre for Stroke and Brain Injury University of Newcastle Callaghan New South Wales Australia; ^4^ Turner Institute for Brain and Mental Health Monash University Melbourne Victoria Australia; ^5^ Centre for Pain IMPACT Neuroscience Research Australia (NeuRA) Randwick New South Wales Australia

**Keywords:** cognitive control, connectivity, EEG, task‐switching, theta

## Abstract

During task‐switching paradigms, both event‐related potentials and time‐frequency analyses show switch and mixing effects at frontal and parietal sites. Switch and mixing effects are associated with increased power in broad frontoparietal networks, typically stronger in the theta band (~4–8 Hz). However, it is not yet known whether mixing and switch costs rely upon common or distinct networks. In this study, we examine proactive and reactive control networks linked to task switching and mixing effects, and whether strength of connectivity in these networks is associated with behavioural outcomes. Participants (*n* = 197) completed a cued‐trials task‐switching paradigm with concurrent electroencephalography, after substantial task practice to establish strong cue‐stimulus–response representations. We used inter‐site phase clustering, a measure of functional connectivity across electrode sites, to establish cross‐site connectivity from a frontal and a parietal seed. Distinct theta networks were activated during proactive and reactive control periods. During the preparation interval, mixing effects were associated with connectivity from the frontal seed to parietal sites, and switch effects with connectivity from the parietal seed to occipital sites. Lateralised occipital connectivity was common to both switch and mixing effects. After target onset, frontal and parietal seeds showed a similar pattern of connectivity across trial types. These findings are consistent with distinct and common proactive control networks and common reactive networks in highly practised task‐switching performers.

## Graphical abstract

During task‐switching paradigms, both event‐related potentials and time‐frequency analyses show switch and mixing effects at frontal and parietal sites. However, it is not yet known whether mixing and switch costs rely upon common or distinct networks. During the preparation interval, mixing effects were associated with connectivity from the frontal seed to parietal sites, and switch effects with connectivity from the parietal seed to occipital sites.

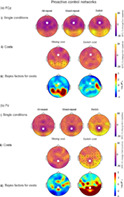

## 1 INTRODUCTION

Cognitive control refers to a group of processes that facilitate goal‐directed adjustments of behaviour, such as overriding automatic responses, set‐shifting, and updating working memory, and involve both common and distinct underlying core mechanisms (Friedman & Miyake, 2017; Miyake et al., 2000). The dual modes of control (Braver, 2012) model posits that these control processes can occur either *proactively* (i.e. planning and preparing in anticipation of a goal) or *reactively* (i.e. adjusting behaviour in response to contextual changes) depending on the context.

Cued‐trials task‐switching paradigms can temporally dissociate between proactive and reactive control processes. In mixed‐task blocks, valid task cues indicate whether to switch or repeat the same task on a trial‐by‐trial basis, and performance is slower and less accurate on switch compared to repeat trials (i.e. switch cost; Rogers & Monsell, 1995). Additionally, performance is slower and less accurate on these repeat trials (i.e. mixed‐repeat) compared to repeat trials in a single‐task block (i.e. all‐repeat trials; Los, 1996). With long cue‐target intervals (CTI), switch and mixing costs are reduced, but not eliminated, leaving residual switch and mixing costs. So, while proactive control processes (e.g. task‐set maintenance and updating) activated during the CTI can reduce costs, reactive control processes that occur after target‐onset appear to be necessary to address target‐driven interference associated with stimulus‐ and response‐level conflict (for review see Karayanidis & McKewen, 2021).

Functional magnetic resonance imaging studies show that frontoparietal networks support cognitive control processes during task switching (Jamadar, Thienel, & Karayanidis, 2015; Ruge, Jamadar, Zimmermann, & Karayanidis, 2013). However, rapid, dynamic adjustments in cognitive control mode, such as the shift from proactive to reactive control, are better characterised using electroencephalography (EEG), which has excellent temporal acuity. Synchronised activity of a local neuronal network can be conceptualised as an electric dipole, and this signal can be recorded from the scalp (e.g. Kappenman & Luck, 2011). A change in the amplitude of an event‐related potential (ERP) component or the power of the time–frequency signal may be indicative of changes within local network properties. In contrast, global network properties may reflect synchronised firing between proximal or distal neural populations, measured using coherence/correlational‐derived metrics (e.g. Cohen, 2014). Critically, such global synchronisation may serve as a communication mechanism whereby information is flexibly routed within networks via oscillatory synchronisation across task or goal‐relevant neural populations (Fries, 2005).

EEG recorded during the cued‐trials task‐switching paradigms can temporally dissociate activity associated with proactive and reactive control processes, and consistently show local frontal and parietal activity. During the CTI, ERPs robustly show a switch‐positivity, a larger cue‐locked centroparietal positivity for *switch* trials than mixed‐repeat trials (Barcelo, Escera, Corral, & Perianez, 2006; Finke, Escera, & Barcelo, 2012; Jost, Mayr, & Rosler, 2008; Karayanidis et al., 2009; Karayanidis, Coltheart, Michie, & Murphy, 2003; Nicholson, Karayanidis, Poboka, Heathcote, & Michie, 2005). Time‐frequency analyses show that, over a similar time window, switch trials elicit higher power over frontal and parietal sites in theta (Cooper, Darriba, Karayanidis, & Barcelo, 2016; Cooper, Wong, McKewen, Michie, & Karayanidis, 2017; Cunillera et al., 2012; McKewen et al., 2020), alpha (8–13 Hz; Cooper et al., 2016; Foxe, Murphy, & De Sanctis, 2014; Mansfield, Karayanidis, & Cohen, 2012), and delta (~0.5–4 Hz; Prada, Barcelo, Herrmann, & Escera, 2014) bands compared to *mixed‐repeat* trials. Additionally, compared to all‐repeat trials, *mixed‐repeat* trials elicit a larger cue‐locked centroparietal positivity, the mixing‐positivity (Jost et al., 2008; Manzi, Nessler, Czernochowski, & Friedman, 2011; Whitson et al., 2014) and higher cue‐locked frontal and parietal theta (Cooper et al., 2017) and alpha (McKewen et al., 2020) power.

After target onset, *switch* trials tend to show a broad negative shift relative to mixed‐repeat trials, resulting in a larger frontal N2 (Goffaux, Phillips, Sinai, & Pushkar, 2006; Jost et al., 2008; Karayanidis, Whitson, Heathcote, & Michie, 2011) and a smaller parietal P3b (Astle, Jackson, & Swainson, 2006, 2008; Jamadar, Hughes, Fulham, Michie, & Karayanidis, 2010; Nicholson et al., 2005) as well as higher frontal theta power (e.g. Enriquez‐Geppert & Barcelo, 2018) than *mixed‐repeat* trials. The centroparietal P3b is also smaller for *mixed‐repeat* than *all‐repeat* trials (Goffaux et al., 2006; Whitson et al., 2014). ERP and theta effects in both proactive (i.e. CTI) and reactive (i.e. post‐target) control periods have been associated with faster RT (e.g. Cooper et al., 2017; Cooper et al., 2019; Karayanidis, Provost, Brown, Paton, & Heathcote, 2011; Karayanidis, Whitson, et al., 2011; Provost, Jamadar, Heathcote, Brown, & Karayanidis, 2018). Although switch and mixing effects span multiple frequency bands, it is the activity in theta that is most often associated with behaviour, so this study focuses on theta activity.

It is not yet known whether these frontal and parietal switch and mixing effects represent distinct cognitive processes or spatially localised activity arising from the same frontoparietal network. Inter‐site phase clustering (ISPC) measures phase similarity between a seed electrode and all other electrodes, providing a way to quantify the presence and strength of connectivity in a particular frequency band. Using a cued‐trial task‐switching paradigm, Lopez, Pusil, Pereda, Maestu, and Barcelo (2019) used ISPC to show that, in the late CTI period, theta and delta frontoparietal connectivity is greater for switch than *repeat* trials. Similarly, in a task‐switching paradigm with no cueing period, Sauseng et al. (2006) found that, after target onset, frontoparietal connectivity was stronger for *switch* than *repeat* trials and this effect was strongest in the theta band. However, these studies focused on either proactive (Lopez et al., 2019) or reactive control only (Sauseng et al., 2006). In a cued‐trials task‐switching paradigm, Cooper et al. (2015) used imaginary coherence to show that distinct frontoparietal networks are engaged during periods that supported proactive versus reactive control. Distinct patterns of theta frontoparietal connectivity were identified during the early CTI period for cues that validly predicted a task switch and after target onset for targets that identified the relevant task. While these findings are consistent with distinct frontoparietal theta connectivity networks for proactive and reactive control, imaginary coherence is not optimal for measuring the strength of this connectivity.

The present study examines the strength of theta connectivity associated with cognitive processes involved in mixing cost and switch cost and activated during the application of proactive versus reactive control. Given robust evidence that frontoparietal networks are involved in proactive and reactive control during task‐switching paradigms, we examine two connectivity networks: one arising from a frontal and the other from a parietal seed. Proactive control processes are captured in the long CTI (1,000 ms), whereas reactive control processes are related to residual control processes that occur after target onset, even after preparation processes have occurred. We also examine whether connectivity strength is associated with interindividual variability in behavioural performance. As, in this paradigm, switch and mixing effects are most prominent in the theta band (McKewen et al., 2020), we focus primarily on theta‐band connectivity.

Given the prior evidence for distinct proactive and reactive control processes in cued‐trials task‐switching paradigms, we expect different patterns of theta connectivity for cue‐locked versus target‐locked ERPs. Within each control period, if the processes that contribute to mixing and switch costs tap into common executive function processes, all‐repeat, mixed‐repeat and switch trials will show a similar distribution of frontoparietal theta connectivity, albeit with progressively increasing strength, as found in ERPs (Karayanidis et al., 2009) and theta power (Cooper et al., 2017). Alternatively, if different processes underlie the switch and mixing costs (McKewen et al. (2020), there will be a distinct pattern of theta connectivity for switch and mixing effects. Finally, we expect that theta connectivity associated with both proactive and reactive control will be correlated with RT, such that increased connectivity in either the proactive or the reactive control periods will be associated with faster responding.

## 2 METHODS

### 2.1 Participants

This study analysed data from the Age‐ility Project (Karayanidis et al., 2016), using the same dataset as McKewen et al. (2020). Age‐ility participants were recruited from a range of sources, including secondary and tertiary education centres, community organisations and local businesses. Two hundred and fifteen participants aged 15–35 years completed the task‐switching paradigm with concurrent EEG. Data from 18 participants were excluded from further analyses: three had very fast RTs and very high error rates suggestive of premature responding; one had an EEG recording problem; 14 had noisy EEG recordings resulting in less than 50 trials for one or more condition/s (Cohen, 2014). This resulted in a final sample of 197 participants, 24 of whom were aged 15–17 years (see Table 1 for demographic information).

**TABLE 1:** Demographic information for participants

Measure	Mean	*SD*
Age (years)	21.41	4.92
Matrix reasoning	54.00	9.44
Span length (forwards)	6.98	1.25
Span length (backwards)	5.13	1.23
Span length (sequencing)	6.73	1.26
Verbal fluency (FAS total)	39.91	9.44
Sex	Males (*N* = 90)	Females (*N* = 107)
Handedness	Right handed (*N* = 180)	Left handed (*N* = 17)

*Note*: There were significantly more females than males (*t*_(196)_ = 43.370, *p* < .001). Matrix reasoning measured with the Wechsler Abbreviated Scale of Intelligence (Wechsler, 1999), span length = number of items recalled during digit span task (Wechsler, 1999).

The protocol complies with the Declaration of Helsinki and was approved by the University of Newcastle Human Research Ethics Committee (HREC: H‐2012‐0157). All participants provided written informed consent (participants under the age of 18 years also provided written parental consent) and were reimbursed $20 per hour. Participants were deemed ineligible for participation if they reported having a chronic psychological or neurological illness, had any contraindication to MRI, or did not have normal to corrected vision. Participants were asked to abstain from caffeine and alcohol at least 2 hr prior to testing.

### 2.2 Stimuli and task

**FIGURE 1:**
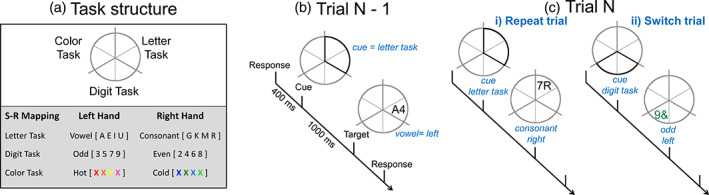
Cued trials task‐switching paradigm. (a) Structure of the task. Adjacent segments are mapped to the colour, letter, or digit task. An example of stimulus–response mapping is also shown. (b) Trial example. A cue highlights two adjacent segments (corresponding here to the letter task) for 1,000 ms. After 1,000 ms, the cue is replaced by a target that appears in one of the highlighted segments. Participants respond to the target and 400 ms after the response the next trial's cue appears. (c) The subsequent trial (N) could be (i) a repeat trial, that is, the same two segments will be highlighted and the same task will be performed, or (ii) a switch trial, that is, the cue will highlight two segments associated with one of the other two tasks and validly indicates which of these tasks the participant will be required to perform on the target

Participants performed a cued‐trials task‐switching paradigm, where they were required to switch between three simple classification tasks: letter (vowel or consonant), number (odd or even) and colour (hot or cold). A grey circle (5° visual angle) divided into six equal‐sized segments was displayed continuously, with a third of the circle associated with a respective task (Figure 1a). These task regions were demarcated with a thicker grey line. Initially, a cue appeared for 1,000 ms, which highlighted two adjacent segments of the circle. The cue was replaced by a target, presented in one of the respective segments just highlighted. The target was comprised of a pair of characters (a letter, a digit or a non‐alphanumerical symbol) presented in either grey or coloured font, thus each target consisted of three dimensions. One dimension is mapped to the currently relevant task (e.g. in Figure 1b, the letter A is a vowel so it is mapped to a left‐handed response), another is mapped to a currently irrelevant task (e.g. in Figure 1b, the number 4 is an even number so it is mapped to a right‐handed response) and the third dimension is neutral and therefore not mapped to any task (e.g. in Figure 1b, the target is shown in grey font). The target remained onscreen until a response was made or a 5,000 ms timeout occurred. The response‐cue interval was 400 ms. The same target never appeared on two consecutive trials.

There are two types of task blocks: single‐task blocks and mixed‐task blocks. Single‐task blocks include only one task per block (i.e. colour, number *or* letter), whereas mixed‐task blocks included trials from all three tasks in a pseudorandom order. Task‐switching trial types were defined by the location of the cue on the current trial (trial *N*) with respect to the preceding trial (trial *N* − 1; Figure 1c). *Repeat* cues highlighted the two segments associated with the task on the prior trial, indicating that the upcoming trial would repeat the same task. *Switch* cues highlighted both segments of a task that was not completed on the prior trial, indicating that the upcoming trial would require a switch in task and identifying which task the participant will be switching to. In single‐task blocks, the cue remained in the same position throughout the block, indicating that the same task was to be *repeated* (*all‐repeat* trials). In mixed‐task blocks, on *mixed‐repeat* trials (25%; Figure 1c‐i), the cue remained in the same position on consecutive trials. Thus, *mixed‐repeat* trials were identical to *all‐repeat* trials but occurred in a mixed‐task block. That is, they were interspersed with *switch* trials (25%; Figure 1c‐ii) on which the cue changed position and highlighted segments associated with one of the other two tasks.

The remaining 50% of trials used partially informative cues and are not used here. *Switch‐away* cues signalled the task would change but did not identify the new task and *noninformative* cues signalled the task may repeat or may switch; see Cooper et al. (2017). As this article focuses on mixing and switch costs, we only report analyses on *all‐repeat*, *mixed‐repeat* and *switch* cues. Each trial type was presented with equal probability in a pseudo‐random sequence so that the same cue type was not repeated on more than four consecutive trials.

Participants were asked to respond as quickly and accurately as possible using their left and right index fingers to press response buttons built into the armrests. The stimulus–response mapping was counterbalanced across participants. An auditory feedback tone was presented following an error. The participant's mean RT and accuracy was displayed at the end of each block and they were encouraged to use this feedback to improve their performance on the next block. A brief rest and a short humorous video (5–10 s) was provided at the end of each block, with a longer break offered mid‐way through the experiment to minimise fatigue.

Participants completed a total of 1,320 practice trials over two training sessions which occurred no more than 14 days apart. After completion of the second training session, participants completed 10 mixed‐task blocks (77 trials/block) and three single‐task blocks (53 trials/block) while EEG was recorded. Each block included five warm‐up trials. The three single‐task blocks (*all‐repeat* trials) always occurred consecutively but were interspersed with the mixed‐task blocks in pseudorandom order.

RT and EEG data analyses were performed on correct trials that (a) had RT between 200 ms and three *SD* from the individual's mean RT, (b) did not follow an error trial and (c) were not the initial five warm‐up trials on each block. On average, 17.96% of trials ±7.67 *SD* were excluded based on these criteria. Trials with high EEG noise levels (see below) were also excluded from RT and EEG analyses. Behavioural data were analysed using two planned comparisons to target mixing cost (*all‐repeat* vs. *mixed‐repeat*) and *switch* cost (*mixed‐repeat* vs. *switch*) using JASP (Version 0.7.5.6; JASP Team, 2020) with a Bonferroni‐corrected significance threshold of *p* < .025 (i.e. *α* = .05/2).

### 2.3 EEG recording and analysis

Continuous EEG was recording using an ActiveTwo Biosemi EEG system (2048 Hz, bandpass filter of DC‐400 Hz) from 64 scalp electrodes plus bilateral mastoids, outer canthi, and both supraorbital and infraorbital ocular sites. Common mode sense and driven right leg electrodes for the Biosemi active electrode system were positioned inferior to P1 and P2, respectively. EEG data were recorded relative to an amplifier reference voltage, and then re‐referenced offline to Cz to remove common‐mode signals. EEG data were processed in MATLAB (Mathworks, Natick, MA) using a custom‐built pipeline utilising Fieldtrip (Oostenveld, Fries, Maris, & Schoffelen, 2011), EEGLab (Delorme & Makeig, 2004), CSD Toolbox (Kayser & Tenke, 2006) and in‐house functions (AW and PC; cf. Cooper et al., 2015). Preprocessing was performed using Fieldtrip as follows: EEG data were re‐referenced offline to electrode Cz, downsampled from 2,048 to 512 Hz using a zero‐phase anti‐aliasing filter with a low‐pass cut off frequency of 245 Hz and then had high pass and notch filtering applied to remove line noise and low‐frequency drift (high pass: 0.1 Hz, forward phase; 50 Hz notch: zero phase). Excessively noisy channels were identified by visual inspection and were excluded. The number of channels deemed bad ranged from 0 to 8, with an average of 0.76 (*SD* = 1.42). To remove blink and vertical eye‐movement artefact, independent components analysis (ICA) was performed using the *fastica* algorithm (Hyvarinen & Oja, 2000). The ICA produced a set of components equal to the number of available electrodes. From this, 1–6 components corresponding to ocular artefact were identified by visual inspection and deleted (mean components = 1.38 ± 0.76 *SD*). Data were then low pass filtered (30 Hz, zero‐phase) to remove high‐frequency noise including muscular artefacts (Table 2).

**TABLE 2:** Mean, *SD* and range for switch and mixing cost error rate, cost RT and cost proportion RT

	Mean (*SD*)	Range
Switch cost error rate	2.37 (3.33)	−2.30–16.77
Mixing cost error rate	0.87 (2.24)	−7.42–8.00
Switch cost RT	160.33 (163.02)	−48.82–1,120.38
Mixing cost RT	115.60 (108.99)	−145.34–579.48
Switch cost proportion RT	0.21 (0.17)	−0.04–0.88
Mixing cost proportion RT	0.19 (0.16)	−0.18–0.79

Trials that contained residual artefact larger than ±120 μV were deleted, resulting in an average of 112.19 (±22.00 *SD*) *all‐repeat*, 134.03 (±25.16 *SD*) *mixed‐repeat*, 128.73 (±27.09 *SD*) *switch* for further analysis. Previously identified bad channels were reintroduced by interpolating data between neighbouring electrodes. EEG data were then transformed using a surface Laplacian filter (smoothing = 10–5, number of iterations = 10, spherical spline order = 4) to reduce volume conduction effects (CSD Toolbox; Kayser & Tenke, 2006).

### 2.4 Time–frequency analysis

Following preprocessing, connectivity analyses were performed on the surface Laplacian filtered data (cf., Cooper et al., 2015) for each cue type (i.e. *all‐repeat*, *repeat* and *switch*). Connectivity was measured using ISPC, which is a time‐resolved and frequency‐band specific, phase‐based measure of functional connectivity between electrodes across trials (Cohen, 2014; Cohen & Gulbinaite, 2014; Gulbinaite, van Rijn, & Cohen, 2014). A phase‐based measure of connectivity was chosen as it is commonly used in the cognitive control literature (e.g. Cohen & van Gaal, 2013; Ryman et al., 2018; van Driel, Swart, Egner, Ridderinkhof, & Cohen, 2015) and has a neurophysiological interpretation. Phase alignment indicates synchronisation of oscillatory processes across two neural populations. When this synchronisation occurs, regions can communicate through neuronal channels open for input and output (Fries, 2005). Although phase‐based analyses are less susceptible to volume conduction than power‐based analyses, we acknowledge that ISPC is not completely insensitive to volume conduction (Cohen, 2014). Thus, analyses were conducted on surface Laplacian filtered data and ISPC was computed as per cent change from baseline (Cohen & Gulbinaite, 2014).

As discussed above, previous cued‐trials task‐switching literature has shown effects of switching and mixing tasks at both frontal and parietal sites. Based on our previous work in the same dataset (McKewen et al., 2020), FCz and Pz were chosen as the seeds for ISPC analyses in order to capture frontal and parietal networks. Based on previous connectivity analyses in cued‐trials task‐switching (Cooper et al., 2015; Lopez et al., 2019; Sauseng, Klimesch, Schabus, & Doppelmayr, 2005), and previous analyses of this dataset (McKewen et al., 2020), we focussed on ISPC analyses on theta activity (defined as 4–8 Hz). McKewen et al. (2020) identified frontal and parietal theta power around 200–500 ms in both post‐cue and post‐target intervals, and we use these same windows to target networks underlying this activity.

ISPC was calculated as follows:

$${\text{ISPC}}_{\mathrm{tf}}=|\frac{1}{N}\times \sum_{n=1}^{N}e^{i\left(\varphi_{j,\mathrm{tf}}-\varphi_{k,\mathrm{tf}}\right)}|$$

where *tf* is one time‐frequency point, *N* is the number of trials, *n* is the trial number, *e* ^*i*^ is the complex component of the exponential, φ is is the phase angle and *k* and *j* are the two electrodes or clusters (cf. Cohen, 2014; Cohen & Gulbinaite, 2014; Gulbinaite, van Rijn, & Cohen, 2014; van Driel et al., 2015). ISPC, therefore, represents the difference in phase angles between two sites or clusters. This subtraction is repeated over trials and averaged to provide a measure of functional connectivity between the seed cluster and each other electrode. ISPC values for each cue type were derived against a 300–100 pre‐cue baseline as a per cent change from baseline.

### 2.5 Statistical analyses

To determine significant effects of switching (*switch* vs. *mixed‐repeat*), and mixing (*mixed‐repea*t vs. *all‐repeat*) tasks, one‐sample *t*‐tests were performed at each electrode (excluding the seed electrode) with false discovery rate (FDR) correction of the level of significance at *α* = .01 (Benjamini, Krieger, & Yekutieli, 2006). This was conducted for each seed (i.e. FCz and Pz) in the CTI and the post‐target period. Correlations between ISPC for all electrodes and RT were analysed for each condition (i.e. *all‐repeat*, *mixed‐repeat*, *switch*) and for each seed during the CTI and post‐target period using Spearman's correlations with FDR correlation of level of significance at *α* = .01. These brain–behaviour correlations were also performed on the proportion costs (i.e. switch‐mixed‐repeat/mixed‐repeat and mixed‐repeat‐all‐repeat/all‐repeat). Given the participants are highly practised and have very low error rates (McKewen et al., 2020) we did not examine the relationship between accuracy and connectivity.

In support of the analysis described above, Bayesian one‐sample *t*‐tests were run to confirm switch and mixing effects and to provide evidence for null effects. The Bayes Factor package (Morey, Rouder, Jamil, & Morey, 2015) in R (R Core Team, 2017) was used with a standard Cauchy prior (*r* scale = 22). This allows us to differentiate between switch‐specific effects and mixing‐specific effects by being able to present direct evidence against the presence of switching effects on mixing trials, and vice versa.

## 3 RESULTS

### 3.1 Behavioural results

**FIGURE 2:**
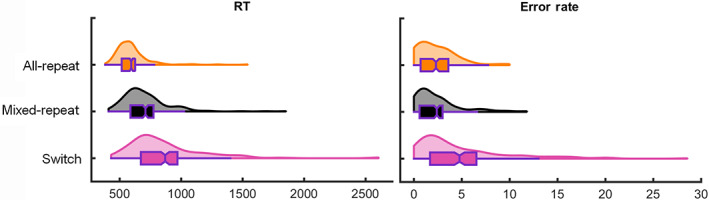
Behavioural task‐switching results. Violin plots showing RT (left) and error rate (right) for each trial type. Plots display the distribution of each data series with a superimposed box and whisker plot. Notch centre is the mean score, box edges = first and third quartile, whisker ends = ±1.5 interquartile range

Behavioural results from this dataset have been published previously (McKewen et al., 2020). Figure 2 and Table 2 shows proportion RT costs produced significant switch (*t*_(196)_ = 17.46, *p* < .001; *d* = 1.24; 95% CI = 0.19, 0.23) and mixing effects (*t*_(196)_ = 16.68, *p* < .001; *d* = 1.19; 95% CI = 0.17, 0.21). Error rate showed a significant switch cost (*t*_(196)_ = 9.99, *p* < .001, *d* = 0.71; 95% CI = 1.90, 2.84) but no mixing cost (*t*_(196)_ = .54, *p* = .59; *d* = 0.04; 95% CI = −0.23, 0.40).

### 3.2 Proactive control networks

**FIGURE 3:**
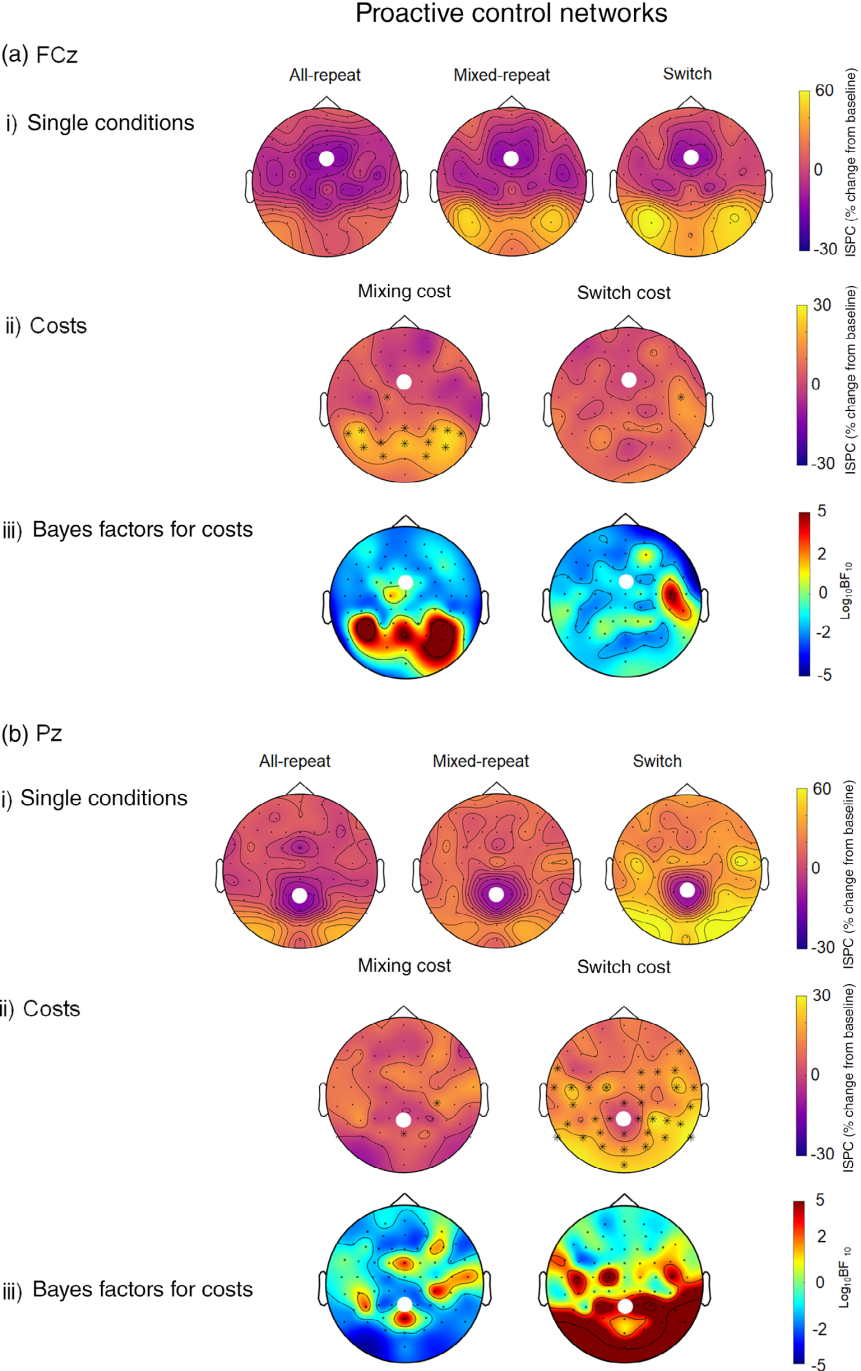
ISPC (per cent change from baseline) in the theta band during the proactive control period (i.e. 200–500 ms after cue onset) between the FCz (a) and Pz (b) seeds and the rest of the scalp for single conditions (i): all‐repeat, mixed‐repeat and switch trials. The seed is indicated by the white dot. The mixing and switch cost plots (ii) show the difference between all‐repeat and mixed‐repeat, and mixed‐repeat and switch trials respectively. Black asterisks indicate a significant difference between conditions at *α* <.01, FDR corrected. Bayesian evidence for the mixing and the switch costs are presented below the cost plots (iii). Using Kass & Rafferty (1995)'s classification, 0–.5 is no evidence, .5–1 is substantial evidence, 1–2 is strong evidence and >2 is decisive evidence. Positive numbers indicate evidence for the alternative (i.e. there is a difference between conditions), while negative numbers indicate evidence for the null (i.e. there is no difference between conditions). ISPC, inter‐site phase clustering

Figure 3a shows ISPC values between FCz and all other electrodes for each condition averaged over 200–500 ms post‐cue (i), and associated mixing and switch cost effects (ii) derived by subtracting the group average ISPC on *all‐repeat* trials from *mixed‐repeat* trials, and *mixed‐repeat* from *switch* trials, respectively. The FCz seed showed the strongest connectivity with bilateral posterior sites centred over PO7 and PO8 electrodes. The strength of connectivity between FCz and lateral parieto‐occipital sites increased across trial types (i.e. from *all‐repeat* to *mixed‐repeat*, and further to *switch* trials). ISPC connectivity strength between the FCz seed and lateral parieto‐occipital sites was significantly greater for *mixed‐repeat* compared to *all‐repeat* trials, indicating a significant mixing cost. In contrast, only a single FCz to right central connection was significantly stronger for *switch* compared to *mixed‐repeat* trials (Figure 3a‐ii).

Figure 3b‐i shows that connections between the Pz seed and bilateral parieto‐occipital and frontocentral sites were most clearly evident for switch trials. The switch cost headplot (Figure 3b‐ii) shows that *switch* trials showed more diffuse connectivity between the Pz seed and widespread centroparietal, parieto‐occipital and frontocentral sites, compared to *mixed‐repeat* trials. In contrast, all‐repeat and mixed‐repeat trials showed similar strength of connectivity between the Pz seed and other sites.

In summary, the FCz seed showed theta coherence suggestive of connectivity between activity at mid‐frontal and parieto‐occipital electrodes primarily for mixing cost (and minimal switch cost effects), whereas the Pz seed showed more diffuse theta connectivity between parietal and parieto‐occipital electrodes primarily for switch cost (and minimal mixing cost effects). For ease of discussion, we refer to these as fronto‐parietal and parieto‐occipital theta connectivity, respectively. However, these are purely descriptive terms and do not infer that they correspond to networks identified by functional MRI activation (e.g. Dosenbach et al., 2006; Dosenbach et al., 2007; Dosenbach, Fair, Cohen, Schlaggar, & Petersen, 2008). This dissociation between networks underlying switch‐specific (parieto‐occipital) and mixing‐specific processes (frontoparietal) was supported by Bayesian analyses (Figure 3a‐iii and b‐iii). There was strong evidence for both the frontoparietal mixing effect and the parieto‐occipital switch effect. Moreover, there was strong evidence in favour of the null for switch effects in fronto‐parietal connectivity and mixing effects in parieto‐occipital connectivity (i.e. log_10_BF_10_ < 0.5).

However, Figure 3 also shows evidence of a common network. Both FCz and Pz seeds showed the strongest theta connectivity with bilateral parieto‐occipital sites. In order to examine this network more closely, we conducted post hoc analyses using seeds at PO7 and PO8, where the effects were strongest and most consistent across trial types.

**FIGURE 4:**
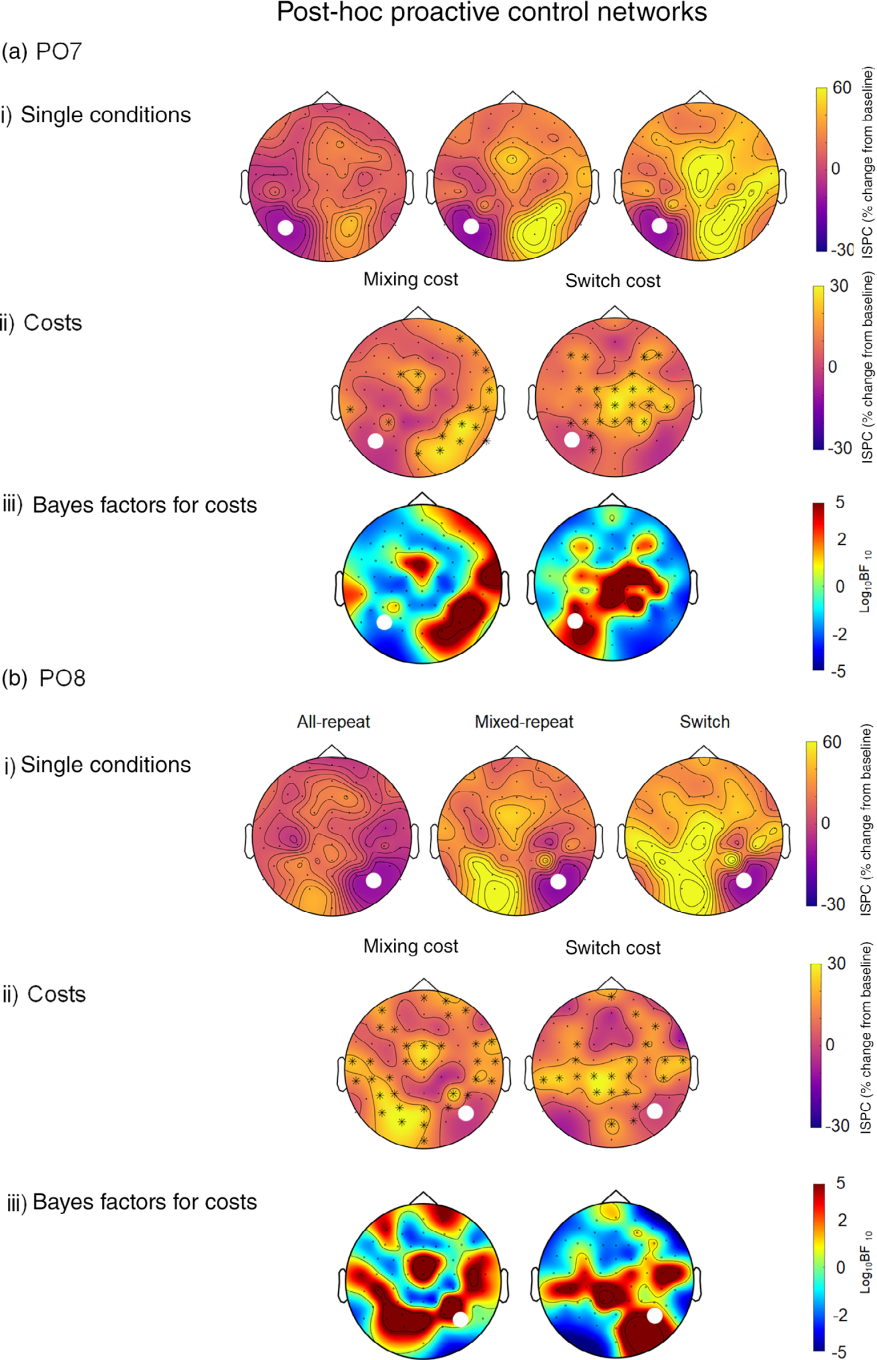
ISPC (per cent change from baseline) in the theta band during the proactive control period (i.e. 200–500 ms after cue onset) between the PO7 (a) and PO8 (b) seeds and the rest of the scalp for single conditions (i): all‐repeat, mixed‐repeat and switch trials. The seed is indicated by the white dot. The mixing and switch cost plots (ii) show the difference between all‐repeat and mixed‐repeat, and mixed‐repeat and switch trials, respectively. Black asterisks indicate a significant difference between conditions at *α* <.01, FDR corrected. Bayesian evidence for the mixing and the switch costs are presented below the cost plots (iii). Using Kass & Rafferty (1995)'s classification, 0–.5 is no evidence, .5–1 is substantial evidence, 1–2 is strong evidence and >2 is decisive evidence. Positive numbers indicate evidence for the alternative (i.e. there is a difference between conditions), while negative numbers indicate evidence for the null (i.e. there is no difference between conditions). ISPC, inter‐site phase clustering

Figure 4 shows connectivity arising from the PO7 seed (a) and the PO8 seed (b). For both *mixed‐repeat* and *switch* trials, the PO7 seed was strongly connected with right parietal sites, spreading anteriorly and laterally, as well as a significant midfrontal connection centred over FCz which was larger for *switch* trials. The mixing cost headplot shows that, compared to *all‐repeat* trials, PO7 theta connectivity for *mixed‐repeat* trials was greater over right parieto‐occipital sites, with a weaker focus frontocentrally (Figure 4a‐ii). The switch cost headplots showed that, compared to *mixed‐repeat* trials, PO7 connectivity was greater for *switch* trials over a frontocentral area spreading from midline into the right hemisphere. A similar pattern of effects was observed for the PO8 seed, where the PO8‐midfrontal network for mixing cost was more prominent (Figure 4b‐ii). This suggests that the FCz‐PO7‐PO8 network may reflect common processes associated with both task switching and task mixing.

**FIGURE 5:**
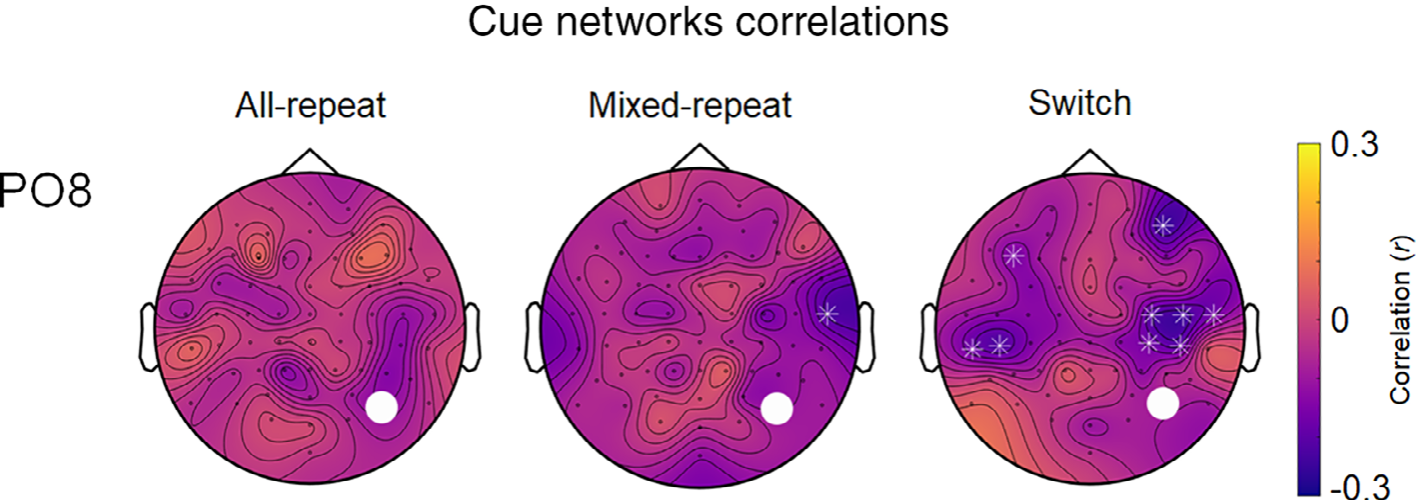
Correlations between RT and ISPC during the proactive control period (i.e. 200–500 ms after cue onset) between PO8 and all other electrodes for all‐repeat, mixed‐repeat and switch trials. The seed is indicated by the white dot. White asterisks indicate a significant correlation between RT and ISPC between PO8 and highlighted electrodes at *α* <.01, FDR corrected. ISPC, inter‐site phase clustering

*Association with response time*: We examined whether ISPC connectivity from FCz, Pz, as well as PO7 and PO8 seeds was associated with response time for the corresponding trial type. Only the PO8 seeded network exhibited any significant correlations with RT (Figure 5). There were no correlations between RT and theta connectivity for *all‐repeat* trials. For *mixed‐repeat* trials, RT was correlated only with the strength of PO8‐C8 connectivity. In contrast, for *switch* trials, RT was correlated with the strength of theta connectivity between PO8 and multiple left and right lateral frontocentral and frontal electrodes (Figure 5). In order to confirm the robustness of this finding, we also tested these correlations at neighbouring electrodes (i.e. PO4, PO3). This resulted in the same pattern of findings—switch trial RT was associated with theta connectivity strength between PO4 and a number of other bilateral anterior electrodes, while the PO3 seed did not show any significant correlations. In all cases, increased ISPC between activity at PO8 and other electrodes was associated with faster RT.

### 3.3 Reactive control networks

**FIGURE 6:**
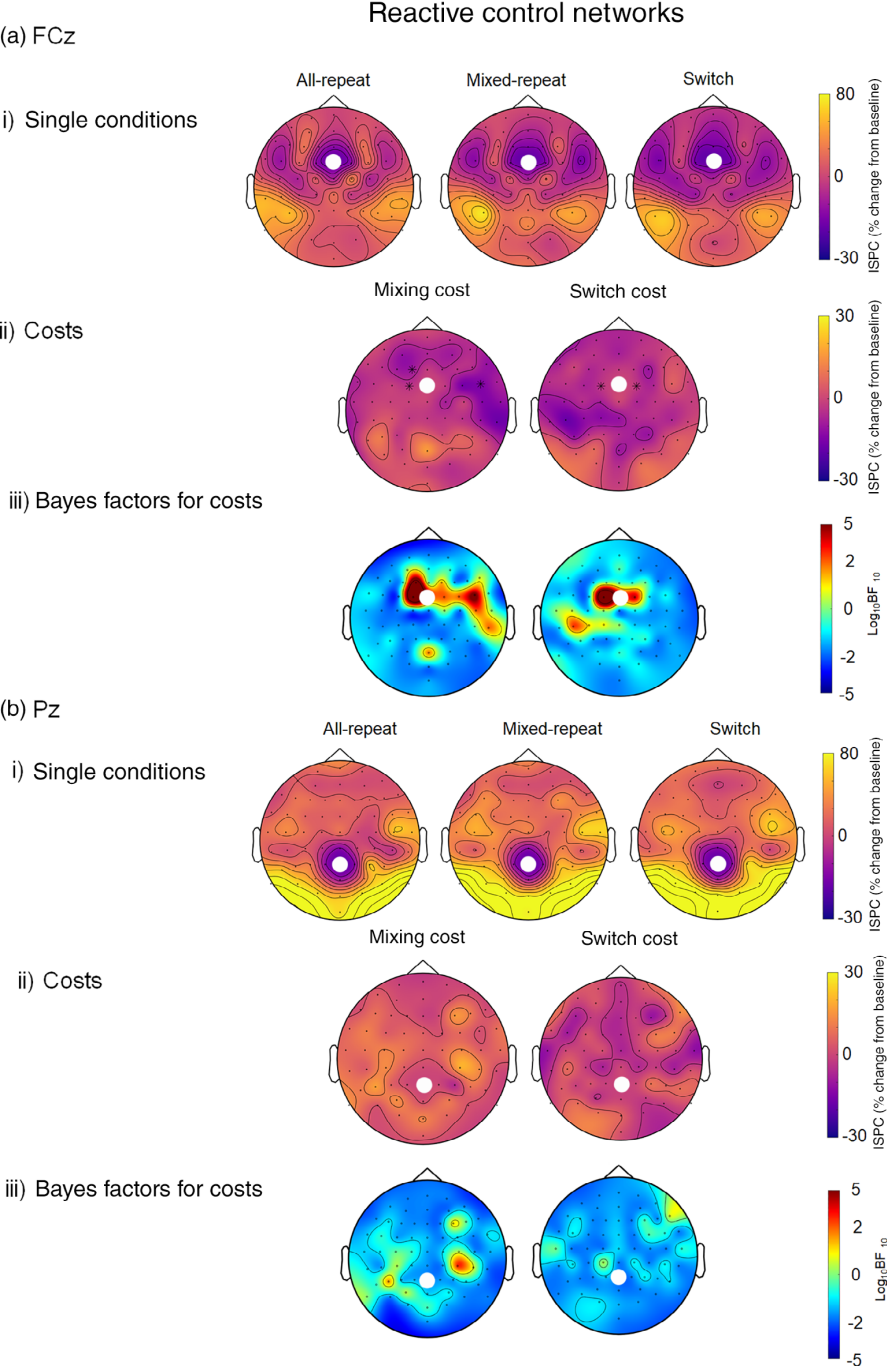
ISPC (per cent change from baseline) in the theta band during the reactive control period (i.e. 200–500 ms after target onset) between the FCz (a) and Pz (b) seeds and the rest of the scalp for single conditions (i): all‐repeat, mixed‐repeat and switch trials. The seed is indicated by the white dot. The mixing and switch cost plots (ii) show the difference between all‐repeat and mixed‐repeat, and mixed‐repeat and switch trials respectively. Black asterisks indicate a significant difference between conditions at *α* <.01, FDR corrected. Bayesian evidence for the mixing and the switch costs are presented below the cost plots (iii). Using Kass & Rafferty (1995)'s classification, 0–.5 is no evidence, .5–1 is substantial evidence, 1–2 is strong evidence and >2 is decisive evidence. Positive numbers indicate evidence for the alternative (i.e. there is a difference between conditions), while negative numbers indicate evidence for the null (i.e. there is no difference between conditions). ISPC, inter‐site phase clustering

Figure 6a,b shows ISPC values between FCz and Pz and all other electrodes after target onset. For all trial types, the FCz seed showed strong ISPC theta connectivity with left parieto‐occipital sites and weaker effects over right parieto‐occipital sites. While these networks appear similar across trial types, mixing and switch cost topography plots show that theta connectivity was weaker for *mixed‐repeat* compared to *all‐repeat* trials, and for *switch* compared to *mixed‐repeat* trials, at proximal frontocentral sites.

Connectivity from the parietal seed (Figure 6b) extended to bilateral parieto‐occipital sites, and more weakly to lateral frontal and central sites. There were minimal switching and mixing effects, with strong evidence for the null (see Figure 6b‐iii).

**FIGURE 7:**
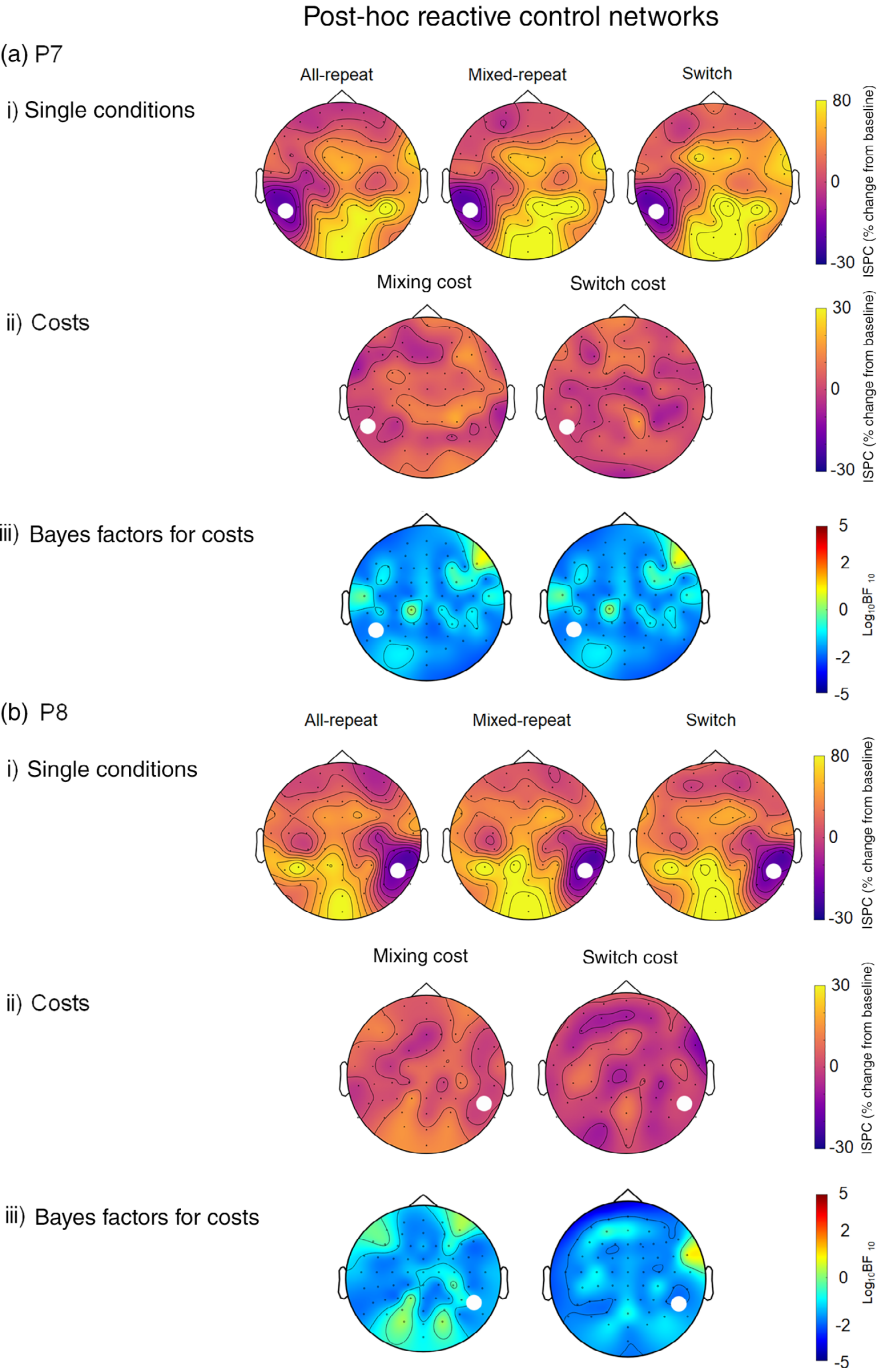
ISPC (per cent change from baseline) in the theta band during the reactive control period (i.e. 200–500 ms after target onset) between the P7 (a) and P8 (b) seeds and the rest of the scalp for single conditions (i): all‐repeat, mixed‐repeat and switch trials. The seed is indicated by the white dot. The mixing and switch cost plots (ii) show the difference between all‐repeat and mixed‐repeat, and mixed‐repeat and switch trials respectively. Black asterisks indicate a significant difference between conditions at *α* <.01, FDR corrected. Bayesian evidence for the mixing and the switch costs are presented below the cost plots (iii). Using Kass & Rafferty (1995)'s classification, 0–.5 is no evidence, .5–1 is substantial evidence, 1–2 is strong evidence and >2 is decisive evidence. Positive numbers indicate evidence for the alternative (i.e. there is a difference between conditions), while negative numbers indicate evidence for the null (i.e. there is no difference between conditions). ISPC, inter‐site phase clustering

Comparing Figures 3a and 6a suggests that connectivity patterns from the frontal seed produced a similar pattern for cue‐locked and target‐locked epochs. However, the post‐target network was somewhat more anterior, peaking most consistently over P7 and P8. We used P7 and P8 as seeds for post hoc ISPC analyses (Figure 7). The target‐locked network seeded at either P7 or P8 showed connectivity with midfrontal and both midline and contralateral parietal sites for all conditions, with no significant mixing or switch.

*Association with response time*: Theta connectivity from the FCz seed to several midline and lateral frontal, central and parietal sites was significantly negatively correlated with RT for the corresponding trial type. Hence, stronger frontal connectivity was associated with faster RT. There were no further correlations in the post‐target networks.

In summary, although the general pattern of frontal and parietal connectivity appeared similar during proactive and reactive control intervals, the former showed dissociable mixing and switch effects, whereas the latter showed minimal trial‐type differences. Moreover, the frontal reactive control network was strongly associated with response time for all trial types. These findings are consistent with a task general network.

## 4 DISCUSSION

Distinct frontoparietal theta networks were found during periods that required proactive (i.e. during the CTI) and reactive (i.e. after target onset) control. Specifically, there were strong distinct connectivity networks associated with mixing and switch costs in the proactive control period (i.e. during the CTI). In contrast, more task‐general frontoparietal theta networks were evident in the reactive control period (i.e. after target onset). Mixing and switch effects in task‐switching have been associated with distinct cognitive control processes (Karayanidis et al., 2010; Kiesel et al., 2010). Mixing cost is attributed to increased working memory load under mixed‐task than single‐task conditions (Los, 1996), whereas switch cost is typically attributed to task‐set reconfiguration (Rogers & Monsell, 1995) as well as interference control processes (Wylie & Allport, 2000).

### 4.1 Proactive control networks

In these highly practised, young participants, mixing and switch effects were stronger during the preparation interval and associated with distinct frontal and parietal networks. The frontal (FCz) seed showed strong parietal‐occipital connectivity that was sensitive to task mixing but not task‐switching effects. Conversely, the parietal (Pz) seed showed strong connectivity with parieto‐occipital, centroparietal and frontocentral sites, which was most evident for switch trials, resulting in sensitivity to task switch but not task mixing effects. Bayesian analyses supported this dissociation, showing strong evidence against switch effects in the frontally seeded network, and against mixing effects in the parietally seeded network. This supports the existence of dissociable networks underlying the switch and mixing costs: connectivity across parietal, occipital, centroparietal and frontocentral was associated with task‐set updating processes, and a network of midfrontal to parieto‐occipital connections associated the maintenance of the relevant task‐set. However, it should be noted that while the FCz‐seeded network showed a mixing cost effect in connectivity between FCz–Pz, the Pz‐seeded network did not show the same effect. This may be due to the conservative *α* < .01 FDR correction we have applied.

In addition, post hoc analyses using the lateral parietoccipital seeds (PO7, PO8) showed a common network of widespread connectivity with parietal, central and frontal locations for both mixing and switch effects. This connectivity pattern is consistent with activation likelihood estimation analysis by Jamadar et al. (2015) who showed that switching tasks (switch > repeat contrasts) are associated with activity in both frontal (e.g. dorsolateral prefrontal cortex, presupplementary motor area, motor cortex) and parietal (e.g. superior and inferior parietal lobule, precuneus) cortex (see Ruge et al., 2013 for review of MRI task‐switching studies). Parietal MRI activation has been shown to be associated with the cue‐locked switch positivity (Jamadar et al., 2010), consistent with the parietal switch‐related network found here.

The proactive control switch network is also largely consistent with strong centroparietal‐posterior theta connectivity during the preparation period using imaginary coherence with the same task‐switching paradigm (Cooper et al. (2015) and ISPC with a different paradigm (Lopez et al., 2019). Despite differences in the time window used to extract these connectivity analyses across these three studies, the consistency in the pattern of results suggests strong involvement of parietal networks with little frontal involvement in task‐set updating, especially in the highly practised participants in this and Cooper's study.

While no studies have examined fronto‐parietal connectivity associated with task mixing effects, our finding of strong fronto‐parietal connectivity for *mixed‐repeat* compared to *all‐repeat* trials is consistent with studies examining localised theta power. During the CTI, mixed‐repeat trials show higher frontal and parietal theta power (Cooper et al., 2017; McKewen et al., 2020) and a larger parietal ERP positivity (e.g. Karayanidis, Whitson, et al., 2011) than *all‐repeat* trials, consistent with activation of a distinct frontoparietoccipital network for mixing effects shown here. This network was not evident for switch cost, indicating a specific role in maintaining the task‐relevant under high interference conditions.

In addition to the above distinct switch‐ and mixing‐related networks, post hoc analyses of PO7 and PO8 seeds showed similar patterns of connectivity with midfrontal, as well as the parietoccipital contralateral site for both mixing and switch contrasts, suggesting a common underlying network. Interestingly, however, the only activity in the PO8‐seeded network was predictive of behaviour response speed, and the network linked with behaviour varied across trial types. For the most difficult *switch* trials, the increased strength of the network linking PO8 with both ipsilateral and contralateral frontal and central locations was associated with faster RT. For *mixed‐repeat* trials, this network was limited to a single connection with an ipsilateral central location, whereas there was no link between behaviour and connectivity for *all‐repeat* trials. These findings suggest that the activation of the PO8 network varies as a function of increased anticipatory allocation of cognitive resources in preparation for processing a more difficult target stimulus.

These findings are consistent with previous work showing a link between theta frontoparietal connectivity and proactive control. Using a cued Simon task, van Driel et al. (2015) found increased theta frontoparietal connectivity for cues associated with incongruent trials compared to cues associated with congruent trials. Similarly, in an AX‐Continuous Performance Task, Ryman et al. (2018) reported increased theta frontoparietal connectivity for rare response switch cues compared to common repeat cues. These findings are consistent with the concept that theta activity reflects an increased need for control (Cavanagh & Frank, 2014; Cavanagh, Zambrano‐Vazquez, & Allen, 2012). Theta frontoparietal connectivity is enhanced in more cognitively demanding trials (e.g. incongruent and switch trials) and this increase in connectivity is associated with improved performance.

It is important to note, however, that while the cued‐trials paradigm used here provides the opportunity for engagement of proactive control period, it is not essential for task performance since target position provides valid information regarding task identity. However, there is now substantial evidence to suggest that participants do use the cue to prepare, even when it is not necessary to do so. Using the same to‐away switching paradigm, previous studies have shown that partially informative (switch‐away cues which indicate that the task will change but do not specify the new task) and non‐informative cues (which indicate that the task may repeat or change) result in longer RT and higher error rate than switch‐to cues (Karayanidis et al., 2009; Mansfield, Karayanidis, Jamadar, Heathcote, & Forstmann, 2011). This same pattern was evident in the present data set (Cooper et al., 2017). These trial types are not reported here as they are not central to the aims of the present article. Furthermore, there is neural evidence of proactive engagement in this task. Cue‐locked midfrontal theta power is associated with the efficiency of preparation, with enhanced theta power for switch trials being associated with smaller RT switch cost (Cooper et al., 2017; Cooper et al., 2019). Finally, in a different cued‐trials task‐switching paradigm, the amplitude of the cue‐locked switch‐positivity is inversely related to RT, so that the larger the switch‐positivity, the faster the response time (Karayanidis, Provost, et al., 2011). These findings are consistent with the activation proactive control processes during the CTI which impact behavioural outcomes.

### 4.2 Reactive control networks

**FIGURE 8:**
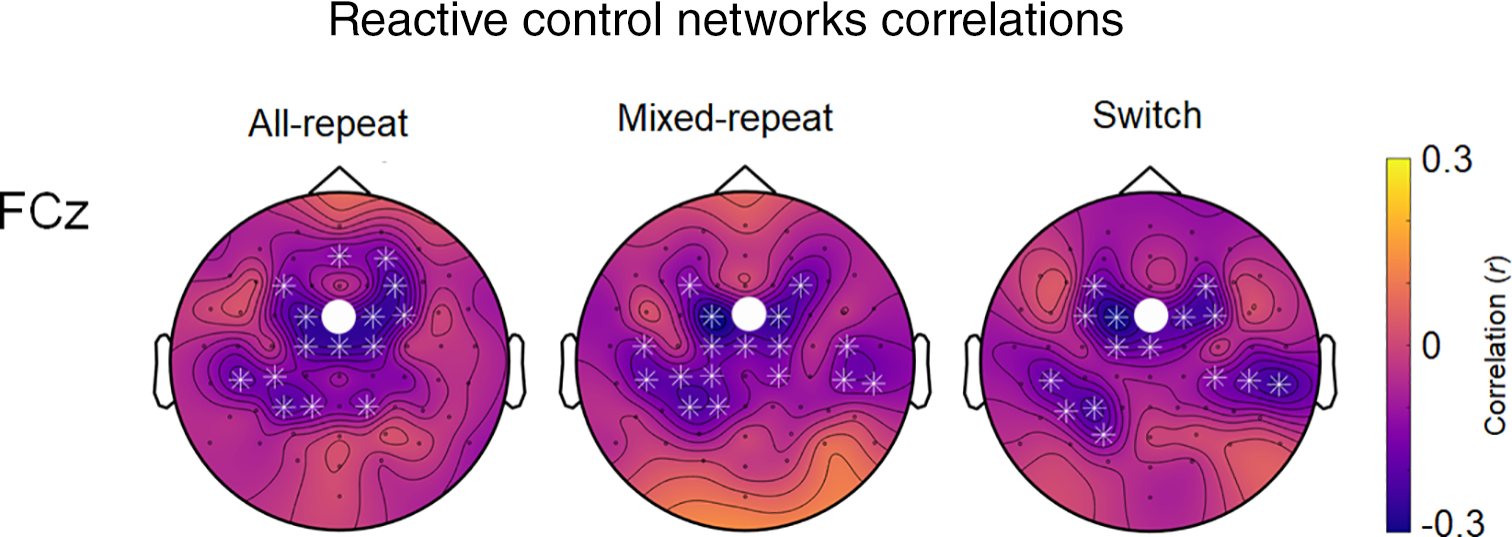
Correlations between RT and ISPC between FCz and all other electrodes for all‐repeat, mixed‐repeat and switch trials. The seed is indicated by the white dot. White asterisks indicate a significant correlation between RT and ISPC between PO8 and highlighted electrodes at *α* <.01, FDR corrected. ISPC, inter‐site phase clustering; FDR, false discovery rate

Reactive control networks seeded at both FCz and Pz also showed strong connectivity with bilateral parietal and parietoccipital sites for all three trial types. However, the strength of these networks did not differ across trial type, resulting in minimal mixing and switch effects on theta connectivity (Figure 6). Higher theta connectivity from the frontal seed to proximal (frontocentral) and more distal (centroparietal) locations was associated with faster RT, and the pattern of correlations was highly consistent across the three trial types (Figure 8).

Similar effects have been observed in time‐frequency power in this same dataset, with no significant switch or mixing effects observed in the post‐target total power (McKewen et al., 2020). With the same paradigm, Cooper et al. (2015) showed no difference in post‐target theta connectivity between fully prepared *mixed‐repeat* and *switch* trials but strong fronto‐parietal connectivity for unprepared switch compared to prepared mixed‐repeat trials. Using a task‐switching paradigm with no opportunity for advance preparation, Sauseng et al. (2006) also showed large post‐target switch effects in theta connectivity. This pattern of findings suggests that highly practised and fully prepared task conditions, reactive control networks are associated with task‐general processes, such as interference control. However, when there is no opportunity for advance preparation (e.g. there is no cue, the cue does not provide task information or the cue‐target‐response mapping is not well established), frontoparietoccipital networks are activated after target onset to complete task‐set updating processes (see Karayanidis et al., 2003, Nicholson et al., 2005 for ERP evidence).

### 4.3 Conclusions

The present study found that the switch and mixing costs in a cued‐trials task‐switching paradigm are reflected in both common and distinct theta connectivity networks in periods conducive to proactive and reactive control. Fronto‐parietal connectivity was involved in setting the goal to switch or repeat task‐set (mixing cost), and parietal‐occipital connectivity was involved in updating task‐set when preparing switch (switch cost). In contrast, there were few differences in theta connectivity between the three cue types in the post‐target period, despite significant behavioural switch and mixing costs. The pattern of correlation between response time and strength of theta connectivity also differed between proactive and reactive control periods. In the preparation interval, a right posterior‐central network was associated with RT for switch trials only, whereas in the post‐target interval, RT was correlated with frontocentral theta connectivity for all three trial types. These findings indicate that, in highly practised individuals and under conditions that allow optimal anticipatory preparation to switch or repeat task, theta connectivity networks arising from frontal and parietal seeds are equally involved in the resolution of target‐driven interference (i.e. stimulus‐level and or response‐level incongruity) on all three trial types. These findings are consistent with distinct mechanisms being associated with goal setting and task‐set updating, as well as with proactive and reactive control processes in task‐switching paradigms (Karayanidis et al., 2010; Karayanidis & Jamadar, 2014).

While this interpretation is consistent with traditional models of task‐switching, alternative approaches to understanding the common and distinct effects theta connectivity effects associated with mixing cost (i.e. goal‐setting) and switch cost (i.e. task‐set updating) may provide more parsimonious interpretations, for example, Friston's free‐energy principle theory (Friston, 2010) and Badre's cortical hierarchies model (Badre, 2008).^1^ In single‐task blocks, all‐repeat trials occur consecutively. Having completed a response to trial *n* − 1, both the cue that indicates the task goal for trial *n*, and the identity of the task‐set to be implemented on trial *n*, are fully predictable (i.e. have no surprise value). During the mixed‐task blocks, the occurrence of mixed‐repeat and switch cues cannot be predicted (i.e. they occur in unique pseudorandom sequences for each block, with no more than four consecutive trials of the same type). For all trials in a mixed‐task block, upon cue onset, participants need to first resolve uncertainty about what to do next (i.e. set the task goal, processes linked to frontal hierarchies) and then use this information to solve uncertainty about how to do it (i.e. maintain the current task‐set, upload the new task‐set, processes liked to parietal hierarchies). Therefore, all trial types in mixed‐task blocks have an additional layer of temporal uncertainty (and, therefore, surprise value) that all‐repeat trials do not. The difference in uncertainty level (i.e. surprise value) between mixed‐repeat versus all‐repeat trials (mixing cost) but not between switch versus mixed‐repeat trials (switch cost) may explain the differential frontoparietal distribution of theta connectivity values for mixing but not switch cost. The lower level mechanisms involved in updating task‐set for switch but not mixed‐repeat trials in mixed‐task blocks may explain the more parietally restricted distribution of theta connectivity for switch versus mixed‐repeat trials (switch cost) but not mixing‐cost. This framework can also account for the lack of differential theta connectivity patterns across the three trial types after target onset. Given the long preparation interval, targets are temporarily predictable events and do not convey much high‐level (frontal) surprise and involve a similar level of S‐R mappings complexity for all trial types. This may explain the similar functional connectivity patterns elicited at target onset, possibly indexing low‐level (parietal) surprise minimisation. So, given highly practised participants and a long preparation interval, different temporal and task contexts influence proactive and reactive control modes.

Finally, while these findings provide strong support for distinct theta connectivity networks associated with proactive control processes involved in maintaining a representation active and updating task‐set, they do not preclude the existence of other proactive control mechanisms that are common to mixing and switch cost. As discussed earlier, task‐switching effects have been observed across multiple frequency bands, and here we focus solely on theta‐band connectivity, and indeed only that arising from two primary seeds. It is possible that other connectivity networks exist in delta, alpha, or beta bands. Moreover, alternative methods for estimating connectivity across the entire network, such as graph theory analyses, may show additional common networks in theta range.

## CONFLICT OF INTEREST

The authors declare that they have no conflict of interest.

## Data Availability

The data that support the findings of this study are available from the corresponding author upon reasonable request.

## Endnote

^1^
We thank an anonymous reviewer for prompting us to consider this interpretation.
